# Microbes, microglia, and pain

**DOI:** 10.1016/j.ynpai.2020.100045

**Published:** 2020-01-29

**Authors:** Zoë Dworsky-Fried, Bradley J. Kerr, Anna M.W. Taylor

**Affiliations:** a Department of Pharmacology, University of Alberta, Edmonton T6G2H7, Canada; b Neuroscience and Mental Health Institute, University of Alberta, Edmonton T6G2H7, Canada; c Department of Anesthesiology and Pain Medicine, University of Alberta, Edmonton T6G2H7, Canada

**Keywords:** Microglia, Gut microbiome, Vagus nerve, Gut permeability, Chronic pain, Gut-brain axis

## Abstract

Globally, it is estimated that one in five people suffer from chronic pain, with prevalence increasing with age. The pathophysiology of chronic pain encompasses complex sensory, immune, and inflammatory interactions within both the central and peripheral nervous systems. Microglia, the resident macrophages of the central nervous system (CNS), are critically involved in the initiation and persistence of chronic pain. Microglia respond to local signals from the CNS but are also modulated by signals from the gastrointestinal tract. Emerging data from preclinical and clinical studies suggest that communication between the gut microbiome, the community of bacteria residing within the gut, and microglia is involved in producing chronic pain. Targeted strategies that manipulate or restore the gut microbiome have been shown to reduce microglial activation and alleviate symptoms associated with inflammation. These data indicate that manipulations of the gut microbiome in chronic pain patients might be a viable strategy in improving pain outcomes. Herein, we discuss the evidence for a connection between microglia and the gut microbiome and explore the mechanisms by which commensal bacteria might influence microglial reactivity to drive chronic pain.

## Highlights

• Explore the connection between the gut microbiome and microglia in chronic pain.
• Discuss mechanisms by which gut bacteria might influence microglia to contribute to chronic pain.
• Highlight gaps in knowledge and discuss future directions for the field.

## 1 Introduction

The transition from acute to chronic pain is defined by numerous adaptations along the entire neural axis. Microglia, the resident immune cells of the central nervous system (CNS), are critically involved in the development and persistence of these adaptations (Beggs et al., 2012, Coull et al., 2005, Inoue and Tsuda, 2009, Sorge et al., 2015, Svensson et al., 2003, Tozaki-Saitoh et al., 2008, Tsuda et al., 2009). While injured or degenerating afferents are clearly involved in driving the inflammatory response to injury in the central nervous system (CNS) (Coull et al., 2005, Guan et al., 2015), emerging evidence suggests that the gut microbiome might contribute to the proinflammatory processes that drive chronic pain.

A relationship between chronic pain and the gut microbiome is becoming increasingly clear. For example, patients with various pain conditions, including visceral pain, chronic pelvic pain, fibromyalgia, and osteoarthritis-related knee pain all display changes in microbiome diversity and abundance compared to healthy individuals (Boer et al., 2019, Braundmeier-Fleming et al., 2016, Minerbi et al., 2019, Nagel et al., 2016, Nickel et al., 2016, Rajilić-Stojanović et al., 2011, Shoskes et al., 2016). Restoring the gut microbiome following dysbiosis improves pain responses in animal models of visceral (Luczynski et al., 2017, O’Mahony et al., 2014, Verdu et al., 2006), inflammatory (Amaral et al., 2008), and neuropathic pain (Ramakrishna et al., 2019, Shen et al., 2017). Although the relationship between pain, microglia, and the gut microbiome has not been directly tested, gut dysbiosis has been implicated in the pathogenesis of several neurological inflammatory conditions, such as depression (Bayer et al., 1999, Jiang et al., 2015, Torres-Platas et al., 2014), multiple sclerosis (Berer et al., 2011, Jack et al., 2005, Mestre et al., 2019, Seifert et al., 2018), and Parkinson’s Disease (Keshavarzian et al., 2015, Sampson et al., 2016, Scheperjans et al., 2015, Sun et al., 2018).

Given the clear connection between the gut microbiome and CNS inflammation, it is possible that perturbations within the community of commensal bacteria contribute to a pathogenic microglial phenotype and facilitate the initiation and maintenance of chronic pain. This review will discuss the complex relationship between the gut microbiome and microglia and explore how intestinal dysbiosis might drive chronic pain through promoting microglial activation.

## 2 Microglial maturation and function is shaped by gut microbes

The gut microbiome regulates the central innate immune system throughout the lifespan, in particular the development and maturation of microglia. Microglia derived from germ-free or antibiotic-treated mice display altered cellular morphologies, lack specific markers of cellular maturity, and have an impaired immune response to immunostimulants (Erny et al., 2015, Matcovitch-Natan et al., 2016). These effects are apparent early in development and are sex-specific, such that male-derived microglia exhibit more differentially expressed genes in germ-free embryos. In adulthood, microglia from germ-free and antibiotic-treated mice also display sex-specific transcriptomic perturbations, particularly in genes related to the immune response (Thion et al., 2018).

Microbial metabolites within the gut, such as short-chain fatty acids, are also involved in regulating the function and maturation of microglia. For instance, mice deficient in free fatty acid receptor (FFAR2) display the same changes in microglial activity as observed in germ-free conditions, and oral administration with short-chain fatty acids restores microglial cell morphology in germ-free animals (Erny et al., 2015). These findings emphasize the necessary role of the gut microbiome in the cellular maturation and proper functioning of microglia.

## 3 How do microglia and gut microbes influence chronic pain?

The role of microglia in the development and transmission of chronic pain is now well-established. Elevated microglial activation and proliferation are observed in animal models of acute, inflammatory, and neuropathic pain (Barcelon et al., 2019, Beggs et al., 2012, Coyle, 1998, Keller et al., 2007, Sweitzer et al., 1999, Tanga et al., 2004, Taylor et al., 2015, Zhang et al., 2005, Zhong et al., 2010). Activated microglia initiate a variety of innate defense mechanisms, including phagocytosis of toxic debris, antigen processing and presentation, and the release of a number of cytokines (Hanisch, 2002, Walter and Neumann, 2009). Production of proinflammatory mediators, such as tumor necrosis factor (TNF)-α and interleukin (IL)-1β, contribute to the activation and sensitization of nerve fibers in the CNS that leads to enhanced pain transmission (McMahon et al., 2005, Watkins et al., 2003). Furthermore, targeted pharmacological interventions that inhibit the activation (Hains and Waxman, 2006, Hua et al., 2005, Raghavendra et al., 2003) or proliferation (Gu et al., 2016) of microglia attenuate neuropathic, inflammatory, and postoperative pain, reinforcing the notion that microglia are integral to pain processes.

**Fig. 1:**
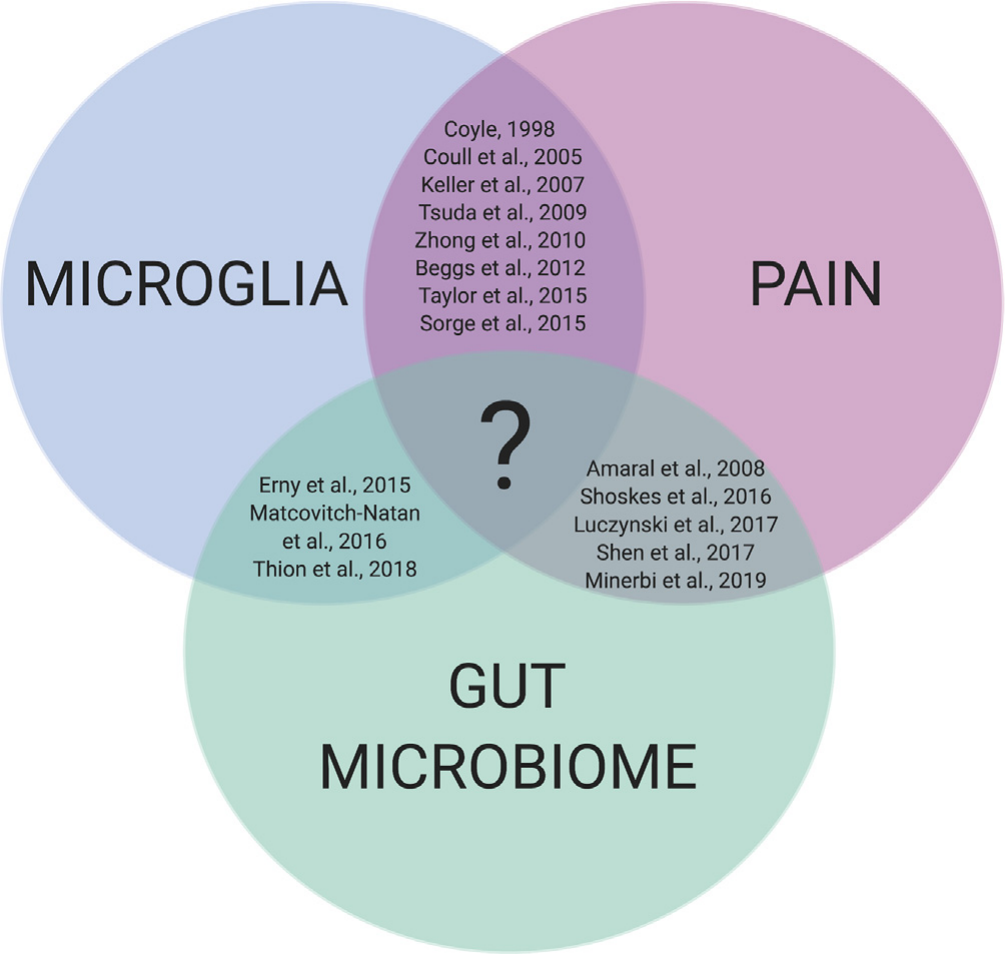
**The intersection between the gut microbiome, microglia, and pain.** There is significant evidence in support of the connections between microglia-pain, microglia-microbiome, and pain-microbiome; however, we currently lack conclusive studies that link the communication between gut bacteria and microglia to the development and maintenance of chronic pain.

Given the established interactions between microglia-pain, microglia-microbiome, and pain-microbiome, it is likely that intestinal dysbiosis and microglial activation are linked in the pathogenesis of chronic pain states (Fig. 1). As an example, the gut microbiome was shown to be the *primary* determinant of pain sensitivity in a model of chemotherapy-induced peripheral neuropathy, and pain sensitivity was significantly correlated with the degree of microglial proliferation in the spinal cord (Ramakrishna et al., 2019). Furthermore, chronic pain observed in patients with complex regional pain syndrome is associated with elevated levels of activated microglia in the spinal cord and brain (Del Valle et al., 2009, Jeon et al., 2017), and reduced gut microbial diversity (Reichenberger et al., 2013). From the work cited above, it is evident that commensal gut bacteria can influence pain responses and microglial cell function. The following section will review the evidence linking the gut microbiome to microglial reactivity in chronic pain and discuss potential mechanisms by which this may occur.

### 3.1 Vagal nerve signaling

The vagus nerve extends from the CNS into the mucosal layers of the gut and serves as the primary bidirectional communication pathway between the gut microbiome and the brain (Forsythe et al., 2014). Considering their close physical proximity, vagal afferent terminals that innervate the gastrointestinal epithelium can interact directly or indirectly with intestinal microbes to influence host physiology at the level of the CNS (Patterson et al., 2002). Bacterial ligands, including toxins and cell wall components, can directly activate nociceptors to produce pain (Chiu et al., 2013, Meseguer et al., 2014). Recent work has demonstrated that particular bacteria taxa, including *Staphylococcus aureus,* activate nociceptors and drive pain via a TRPV1 channel-dependent mechanism (Blake et al., 2018). Cells within the nodose ganglia of the vagus nerve express TRPV1 (Kupari et al., 2019); therefore, it is conceivable that certain bacterial species within the gut can act directly on vagal afferents and contribute to pain pathogenesis. Moreover, local infection in the gut with the pathogen *Campylobacter jejuni* is sufficient to induce expression of neuronal activation marker c-FOS in vagal sensory neurons (Goehler et al., 2005). Administration of bacteria *Lactobacillus rhamnosus* reduces anxiety- and depression-like behaviour in mice, and this effect is abated following vagotomy (Bravo et al., 2011). These findings demonstrate the involvement of the vagus nerve in the relay of information from the gut to the brain, which likely influences inflammation within the CNS.

Indeed, communication between bacteria within the gut and vagal afferents is implicated in modulating central inflammation. Vagal nerve afferents express cytokine receptors, allowing the vagus nerve to sense changes in the inflammatory state of the gut and relay these signals to the CNS (Ek et al., 1998, Goehler et al., 1999). Microglial reactivity and morphology are also influenced by vagal activity. Stimulation of the vagus nerve dampens microglial proliferation and the expression of proinflammatory cytokines following lipopolysaccharide (LPS)-induced inflammation (Meneses et al., 2016), and enhances microglial cell ramification in naive mice (Kaczmarczyk et al., 2018). The vagus nerve may therefore serve as a major anatomical pathway for signals derived from intestinal microbes to influence microglial activation, and in turn contribute to pain transmission.

### 3.2 Gut permeability

A pertinent function of the gut microbiome is the development and maintenance of the intestinal barrier, which separates luminal bacteria and pathogens from the underlying immune cells (Ohland and MacNaughton, 2010). The intestinal barrier is composed of a protective mucosal layer and a monolayer of intestinal epithelial cells interconnected by tight junctions. Under normal, physiological conditions, translocation of microbes and microbial components is limited by this barrier. Commensal bacteria protect the integrity of the epithelial barrier by inhibiting the colonization of pathogens on mucosal surfaces and the invasion of these microorganisms into epithelial cells and the circulation (Tlaskalová-Hogenová, 2004, Turner, 2009). For instance, mice with intestinal dysbiosis exhibit increased permeability of the epithelial barrier (Rocha et al., 2014). Indigenous gut microbes further influence epithelial barrier permeability indirectly through communication with host immune cells (Arrieta et al., 2006). Finally, microbial-derived short-chain fatty acids, such as butyrate and acetate, preserve intestinal barrier integrity by maintaining epithelial tight junction proteins (Kelly et al., 2015).

Disrupted barrier function and increased permeability of the epithelium are associated with changes in the composition of the gut flora (Bansal et al., 2010, Fukuda et al., 2011). Breakdown of the intestinal barrier (leaky gut) due to bacterial dysbiosis permits leakage of neuroactive microbial compounds and immune products across the intestinal wall and into the systemic circulation, influencing peripheral inflammation (Crumeyrolle-Arias et al., 2014, Kelly et al., 2015).

Increases in intestinal permeability are associated with elevated plasma levels of the proinflammatory cytokines TNF-ɑ and IL-6 (Cariello et al., 2010). Initiation of a peripheral immune response can contribute to central inflammation, including microglial activation. This might happen directly, given that immune cells and certain cytokines (e.g. TNF-ɑ, IL-1) in the circulation can directly cross the blood-brain barrier (BBB) and activate microglia (Banks and Kastin, 1991, Gutierrez et al., 1993). This peripheral to central immune communication might be further facilitated by a breakdown in the BBB. Indeed, increased peripheral inflammation is associated with disrupted BBB integrity and central inflammation (Varatharaj and Galea, 2017, Zhu et al., 2018). Enhanced permeability of the BBB allows small molecule bacterial components and metabolites to enter the CNS and trigger the aberrant activation of microglia (Qin et al., 2007). In fact, manipulation of the gut microbiome has been shown to compromise BBB integrity. Fetal and adult germ-free mice display increased BBB permeability and altered brain expression of tight junction proteins (Braniste et al., 2014). This loss of BBB integrity following manipulation of the gut microbiome likely contributes to heightened microglial activation and central inflammation.

Disrupted intestinal barrier integrity has been reported in several chronic pain conditions, including fibromyalgia, complex regional pain syndrome, and irritable bowel syndrome (Goebel et al., 2008, Piche et al., 2009). The degree of intestinal permeability is associated with pain severity (Piche et al., 2009) and plasma levels of several proinflammatory cytokines, such as IL-2, IL-6, and TNF-α (Ernberg et al., 2018). These studies support the hypothesis that leaky gut leads to systemic and central inflammation and contributes to the pathophysiology of chronic pain.

It is unclear whether breakdown of the intestinal barrier in diseases and disorders associated with chronic pain is a cause or consequence of the pain response. It is likely a bidirectional mechanism whereby gut dysbiosis leads to leaky gut and drives the initiation of chronic pain, further disrupting gut homeostasis and the microbial community. This mechanism needs to be investigated in preclinical and clinical studies to establish the influence of gut microbes in this relationship. Such studies will identify if targeting the intestinal barrier is a viable strategy in treating chronic pain.

### 3.3 Signaling mechanisms

The previous section reviewed system-level mechanisms by which gut microbes can influence microglial activation in chronic pain states. Emerging evidence has also identified precise signaling mechanisms and molecules that might further contribute to these pathways. This evidence will be highlighted in the following sections.

#### 3.3.1 TLR4-mediated signaling

Signaling via toll-like receptors (TLRs), a class of pattern recognition receptors, plays a key role in the innate immune system and sensory processing (Lacagnina et al., 2018, Nicotra et al., 2012). In particular, activation of TLR4s on spinal microglia stimulates an inflammatory signaling cascade that leads to the production of proinflammatory cytokines and contributes to pain hypersensitivity (Saito et al., 2010, Shimazu et al., 1999, Tanga et al., 2005).

Bacteria-derived LPS can also directly activate and sensitize trigeminal and dorsal root ganglion sensory neurons via TLR binding (Diogenes et al., 2011, Qi et al., 2011). LPS derived from gram-negative bacteria binds TLR4 expressed on microglial cells and promotes microglial activation and the production of proinflammatory molecules (Clark et al., 2010, Lehnard et al., 2002, Saito et al., 2010). TLR4 signaling might become more prominent following intestinal dysbiosis. For example, inflammatory bowel disease is associated with increased expression of TLR4 in humans (Szebeni et al., 2008), possibly contributing to the pathological inflammation associated with chronic pain. Activation of TLR4 on immune cells may also signal back to the gut in a reciprocal manner to influence intestinal dysbiosis and pain processing. In a murine model of chemotherapy-induced peripheral neuropathy, loss of TLR4 expression improves intestinal function and reduces pain responses (Shen et al., 2017, Wardill et al., 2016). These findings support an essential role for the intestinal microbiome in the production of chronic pain through an LPS-TLR4 dependent pathway.

#### 3.3.2 Cytokines

Inflammatory cytokines in the central and peripheral nervous systems are critical in the initiation and persistence of several pathological pain states (Cunha et al., 1992, DeLeo et al., 1996, DeLeo et al., 1997, Perkins and Kelly, 1994, Ramer et al., 1998). There is now evidence to suggest that gut bacteria can influence levels of circulating cytokines and microglial reactivity. Luczynski et al. (2017) found that germ-free mice display elevated transcription levels of the proinflammatory cytokines IL-6, IL-β, and TNF-ɑ in the spinal cord compared to conventionally colonized mice. Increased expression of proinflammatory cytokines in germ-free mice correlated with heightened microglial activation, visceral hypersensitivity, and TLR upregulation. These symptoms were normalized to control levels following microbial colonization. In contrast, Erny et al. (2015) showed that germ-free conditions lead to decreased microglial reactivity and cytokine release. These conflicting results may be attributed to methodological discrepancies, such as the use of different mouse species. It is interesting to note that Luczynski et al. (2017) performed experiments using only male mice, whereas Erny et al. (2015) used a mixed-sex cohort. In consideration of the sex-dependent function of microglial cells (Crain et al., 2013, Sorge et al., 2015, Taves et al., 2016, Weinhard et al., 2018), perhaps these findings are inconsistent because there is a sex-specific mechanism that drives inflammation in response to microbiome depletion. In any case, these studies clearly indicate a role for the gut microbiome in cytokine-mediated signaling, though the direction of this influence is unclear. Further studies using germ-free animals are required to confirm how the gut microbiome alters microglial reactivity and cytokine levels, and how this might differ between the sexes.

Anti-inflammatory cytokines are immunoregulatory molecules that dampen the proinflammatory response. Among the anti-inflammatory cytokines, IL-10 produced by innate immune cells, including microglia, is involved in combating damage driven by excessive inflammation (Moore et al., 2001). The link between anti-inflammatory cytokines, the gut microbiome, and pain is robust. To illustrate, germ-free mice produce greater amounts of IL-10 as compared to mice with a conventional microbiome (Souza et al., 2004). In humans, IL-10 plays an essential role in diminishing gut inflammation (Braunstein et al., 1997, Schreiber et al., 1995). Patients with irritable bowel syndrome who present improvements in abdominal pain following treatment with oral probiotics also display increased levels of IL-10 (O’Mahony et al., 2005).

IL-10 has further been shown to influence non-visceral pain. Administration of IL-10 suppresses the development of neuropathic and inflammatory pain in diverse experimental models, including complex regional pain syndrome, peripheral nerve injury, spinal nerve ligation, and multiple sclerosis (Grace et al., 2017, Kim et al., 2018, Lee et al., 2013, Milligan et al., 2005). Upon exposure to external inflammatory mediators, germ-free mice exhibit enhanced expression of peripheral IL-10 and diminished hyperalgesia and allodynia (Amaral et al., 2008). Treatment with an anti-IL-10 antibody produces pain hypersensitivity in germ-free mice, suggesting an important interaction between commensal gut bacteria and the host immune system in inflammatory pain. As the gut microbiome plays an essential role in the regulation of the immune response via cytokine signaling, intestinal microbes might similarly modulate cytokine-mediated pain processes through interacting with microglia.

#### 3.3.3 BDNF

Brain-derived neurotrophic factor (BDNF) is a neurotrophin involved in neuronal survival, differentiation, neurogenesis, and the regulation of emotional and cognitive behaviours. While the cellular origin of BDNF is highly debated, as BDNF has been observed in sensory neurons (Dembo et al., 2018, Yu et al., 2020), astrocytes (Parpura and Zorec, 2010), and microglia (Coull et al., 2005, Trang et al., 2011), the role as an important signaling molecule in neuropathic pain transmission is well-appreciated (Coull et al., 2005, Keller et al., 2007). Dysregulation of intestinal microbiome composition might influence BDNF release in the spinal cord and contribute to the genesis of pain. For example, a recent study demonstrated that in a rat model of visceral hypersensitivity, treatment with the probiotic *Lactobacillus plantarum* attenuates visceral pain responses during colorectal distension and reduces spinal BDNF expression (Liu et al., 2019).

A number of studies that employ different techniques to manipulate microbiome composition suggest that BDNF expression in various brain regions, including the amygdala and hippocampus, is highly sensitive to perturbations of the gut microbiome (Arentsen et al., 2015, Bercik et al., 2011, Desbonnet et al., 2015, Gareau et al., 2011, Neufeld et al., 2011, Sudo et al., 2004). Impairments in cognitive functioning and mood due to intestinal dysbiosis are associated with alterations in BDNF expression (Bercik et al., 2011, Desbonnet et al., 2015, Gareau et al., 2011). Similarly, it is hypothesized that disruptions to microglial homeostasis, such as an increase or decline in activity, underlie depression (Yirmiya et al., 2015). As both chronic pain and gastrointestinal symptoms are often comorbid with disorders of affect, including depression and anxiety, it is possible that the interplay between the microbiome, microglia, and BDNF extends beyond mood disorders to pain conditions (Lerman et al., 2015, Mussell et al., 2008).

It is worth noting that the requirement for BDNF-mediated signaling in the development and maintenance of chronic pain appears to be sex-specific. Inhibition of BDNF signaling in the spinal cord reverses mechanical allodynia in male but not female mice, and males lacking microglial BDNF fail to develop allodynia as displayed by female and wildtype mice (Sorge et al., 2015). To date, no study has examined sexual dimorphisms in the relationship between the gut microbiome and BDNF, thus this remains an important area for further study.

## 4 Future perspectives and challenges

Microglia have long been known to play an integral role in the initiation and maintenance of chronic pain. In preclinical studies, inhibiting microglia is effective in treating chronic pain (Hains and Waxman, 2006, Ledeboer et al., 2005, Meller et al., 1994, Watkins et al., 1997). Given the robust preclinical evidence, one might expect microglial inhibitors to be widely adopted clinically. However, a single specific microglial inhibitor has yet to be developed for the treatment of chronic pain. Some of this translational failure may be due to the challenges of reversing microglial activation once it has already been established. In addition, success has been limited by the challenge of developing specific, brain-penetrating, and safe therapeutics that effectively inhibit microglia. As such, there remains a large unmet clinical need to design alternative or superior strategies to alter microglial reactivity in order to capitalize on the promise suggested in preclinical literature.

The advances in our understanding of the interplay between intestinal microbes and microglia have been tremendous in recent years. Converging lines of evidence from preclinical and clinical studies support the prevailing hypothesis that the microbiome and microglia communicate to modulate brain health and disease. It has now been firmly established that strategies that manipulate or restore the gut microbiome are effective at reducing microglial activation and improving symptoms associated with inflammation. The purpose of this review article is to draw attention to the emerging evidence linking gut dysbiosis with microglial activation in chronic pain. These data suggest that strategies that target gut health in chronic pain show great promise in improving pain outcomes in chronic pain populations, though much more research is needed.

**Fig. 2:**
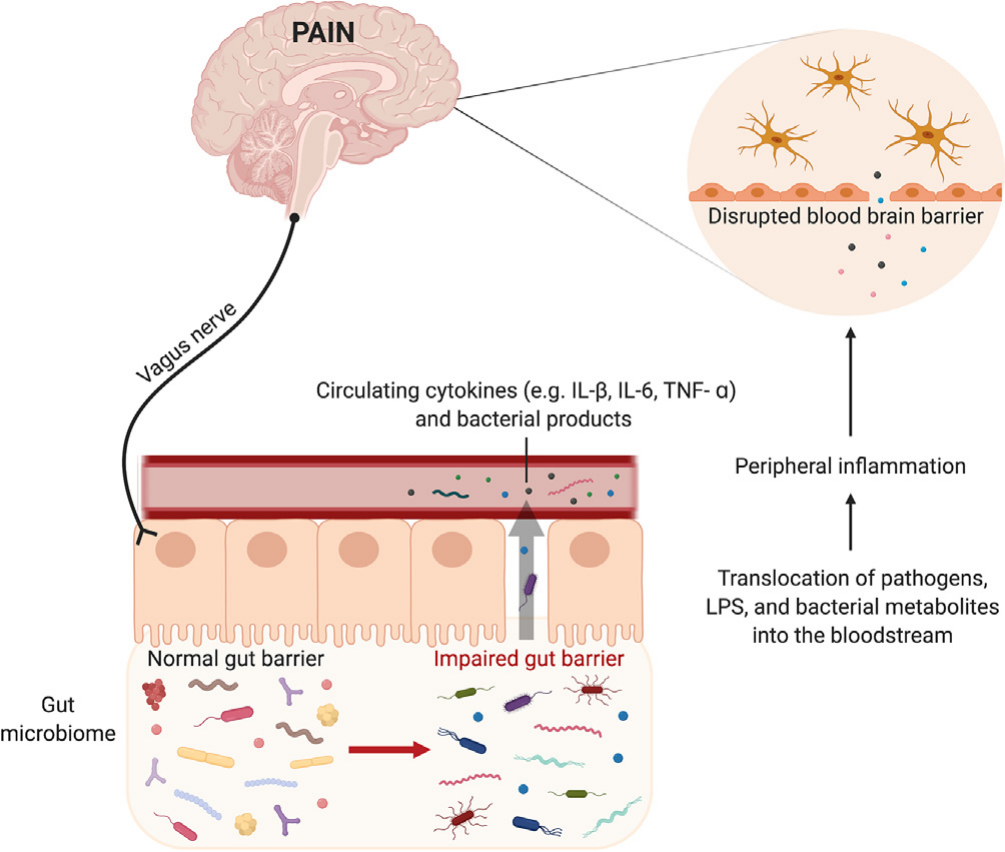
**Mechanisms by which the gut microbiome might influence microglial activation to drive chronic pain.** There are numerous mechanisms through which gut bacteria might influence microglial reactivity to drive the initiation and maintenance of chronic pain. Bidirectional signaling between gut bacteria and the brain via the vagus nerve plays a role in modulating microglial proliferation and activation. Impaired gut barrier function permits leakage of bacterial products into the systemic circulation, causing a peripheral immune response and subsequent microglial activation. Cytokines and immune cells can activate microglia either by directly crossing the intact BBB or through regions of enhanced permeability. Through these routes, microglia are activated and contribute to the production of chronic pain. *LPS: lipopolysaccharide; BBB: blood brain barrier.*

One outstanding question is the precise mechanism by which the gut microbiome influences microglia in pain states. Here, we review several possible mechanisms, including vagal nerve signaling, disruptions to the gut epithelial barrier, and circulating bacterial metabolites (Fig. 2). However, firmly identifying the mechanism (or mechanisms) by which gut microbes influence microglia will be necessary to develop safe and effective strategies to interfere with gut-microglial interactions in chronic pain. Despite exciting preclinical results demonstrating that manipulation of the gut microbiome alters pain sensitivity and microglial activation, it is important to consider the translational capacity and relevance of controlled preclinical conditions, such as germ-free mice, to human physiology. These preclinical animal studies should be interpreted cautiously and further investigations testing this hypothesis in human populations are needed.

Nevertheless, the growing body of literature provides support for the idea that the gut microbiome contributes to central inflammation and chronic pain pathology. It is important to note that the mechanisms explored are necessarily speculative, as there is a paucity of studies that conclusively link pain responses to microbiome-mediated alterations in the gut and central inflammation. Further investigations are imperative to gain mechanistic insight into how gut bacteria and microglia communicate to produce pain and to understand the therapeutic potential of manipulating the microbiome for pain relief and disease.

## Funding sources

Financial support was provided by a Canada Graduate Scholarship from the Canadian Institutes of Health Research (ZDF).

## Declaration of Competing Interest

The authors declare that they have no known competing financial interests or personal relationships that could have appeared to influence the work reported in this paper.
